# How a point-of-care dashboard facilitates co-production of health care and health for and with individuals with psychotic disorders: a mixed-methods case study

**DOI:** 10.1186/s12913-022-08992-2

**Published:** 2022-12-30

**Authors:** Andreas Gremyr, Christopher Holmberg, Johan Thor, Ulf Malm, Boel Andersson Gäre, Ann-Christine Andersson

**Affiliations:** 1 Department of Psychotic Disorders, Sahlgrenska University, Hospital, Sahlgrenska Universitetssjukhuset Psykiatri Psykos, Göteborgsvägen 31, 431 80, Mölndal, Sweden; 2 Jönköping Academy for Improvement of Health and Welfare, School of Health and Welfare, Jönköping University, Barnarpsgatan 39, 55111 Jönköping, Sweden; 3 Institute of Health and Care Sciences, University of Gothenburg, Arvid Wallgrens Backe, Box 457, 405 30 Göteborg, Sweden; 4 Sahlgrenska Academy at Gothenburg University, Institute of Neuroscience and Physiology, Box 400, 40530 Göteborg, Sweden; 5 Futurum Academy for Health and Care, Region Jönköping County, Jönköping, Sweden; 6 Department of Care Science, Malmö University, Nordenskiöldsgatan 1, 21119 Malmö, Sweden

**Keywords:** Coproduction, Learning health systems, Schizophrenia, Psychosis

## Abstract

**Background:**

Individuals with psychotic disorders experience widespread treatment failures and risk early death. Sweden’s largest department specializing in psychotic disorders sought to improve patients’ health by developing a point-of-care dashboard to support joint planning and co-production of care. The dashboard was tested for 18 months and included more than 400 patients at two outpatient clinics.

**Methods:**

This study evaluates the dashboard by addressing two questions:

1. Can differences in health-related outcome measures be attributed to the use of the dashboard?
2. How did the case managers experience the accessibility, use, and usefulness of the dashboard for co-producing care with individuals with psychotic disorders?

This mixed-method case study used both Patient-Reported Outcome Measures (PROM) and data from a focus group interview with case managers. Data collection and analysis were framed by the Clinical Adoption Meta Model (CAMM) phases: i) accessibility, ii) system use, iii) behavior, and iv) clinical outcomes. The PROM used was the 12-item World Health Organization Disability Assessment Schedule (WHODAS 2.0), which assesses functional impairment and disability. Patients at clinics using the dashboard were matched with patients at clinics not using the dashboard. PROM data were compared using non-parametric statistics due to skewness in distribution. The focus group included five case managers who had experience using the dashboard with patients.

**Results:**

Compared to patients from clinics that did not use the dashboard, patients from clinics that did use the dashboard improved significantly overall (*p* = 0.045) and in the domain self-care (*p* = 0.041). Focus group participants reported that the dashboard supported data feedback-informed care and a proactive stance related to changes in patients’ health. The dashboard helped users identify critical changes and enabled joint planning and evaluation.

**Conclusion:**

Dashboard use was related to better patient health (WHODAS scores) when compared with matched patients from clinics that did not use the dashboard. In addition, case managers had a positive experience using the dashboard. Dashboard use might have lowered the risk for missing critical changes in patients’ health while increasing the ability to proactively address needs. Future studies should investigate how to enhance patient co-production through use of supportive technologies.

**Supplementary Information:**

The online version contains supplementary material available at 10.1186/s12913-022-08992-2.

## Introduction

The quality of Swedish healthcare is praised in numerous reports [1, 2], but care and outcomes in a national cohort of persons with psychotic disorders (e.g., schizophrenia) (*n* = 29,823) are marked by widespread treatment failures (> 70%), reflected by suicide attempts, discontinuation of or switch to other medication, or death [3]. Schizophrenia is a chronic severe mental illness (SMI) accounting for 1.1% of Disability Adjusted Life Years (DALYs) lost worldwide [4], with a point prevalence of 4.6 of 1000 persons globally [5]. No or discontinued treatment of schizophrenia greatly increases the hazard ratio for early death, leading to excess mortality, especially evident among first-episode patients [6]. Patient participation in care–i.e., being able to make informed decisions on the course of treatment–is considered essential [7]. Furthermore, Sweden’s Patient Act requires that patients be given the opportunity to participate in their own care [8]. Co-production of healthcare services represents “the interdependent work of users and professionals who are creating, designing, producing, delivering, assessing, and evaluating the relationships and actions that contribute to the health of individuals and populations” ([9] p.2). Efforts to involve patients [10, 11] have been based on considering ethical issues [12, 13], mobilizing patients’ resources [14], and increasing involvement to improve outcomes and lower costs [15, 16]. Co-production reflects societal trends in favor of more active and influential roles for service users [17]. Recently, technology has been seen as a way to increase patient co-production of care at scale in Learning Health Systems (LHS); for example, data visualizations (i.e., dashboards) have been used jointly by patients and clinicians at point-of-care (PoC) [10, 18–20].

Sweden’s largest department specializing in psychotic disorders is in Gothenburg’s Sahlgrenska University Hospital. This department seeks to improve patients’ health by enabling a more data-driven approach to services for persons with psychotic disorders. Healthcare professionals identified the need to plan patients’ health services jointly with patients during visits and to assess and review progress using a user-friendly interface. Therefore, a PoC data dashboard [21] was developed to incorporate the patient’s perspective such as Patient-Reported Outcome Measures (PROM) and the clinical perspective (a symptom/remission scale) with data available from health information systems such as the Electronic Health Record (EHR). The dashboard was designed to support patient co-production of treatment and therefore achieve better health and care for people with psychotic disorders and other severe mental illnesses.

## Methods

This paper reports an evaluation of an initiative to deploy and test the dashboard in the clinical (pilot) setting. The aims were to assess the dashboard’s utility and contribution to improving health for people with schizophrenia by addressing the following evaluation questions:

3) Can differences in health-related outcome measures be attributed to the use of the dashboard?
4) How did the case managers experience the accessibility, use, and usefulness of the dashboard for co-producing care with individuals with psychotic disorders?

### A case study with a mixed methods design

This mixed-method case study combines quantitative data from the dashboard and qualitative data from a focus group interview with healthcare professionals. Specifically, this study evaluated changes in clinical and patient outcomes derived from PROMs related to the use of the dashboard. To report this study in a systematic and transparent way, we used the Good Reporting of a Mixed Methods Study (GRAMMS) checklist. This guideline suggests providing the rationales for using the approach, the sampling techniques, the sequencing, the priorities, the integration of data, and analysis techniques (Appendix 2) [22].

### Context

The department delivers secondary and tertiary care for people with psychotic disorders, schizophrenia being the most common diagnosis. Between 2700 and 3000 patients are offered specialized care at seven outpatient clinics in the metropolitan Gothenburg area (a population of approximately 600,000 people). A fifth of these patients need psychiatric inpatient care each year, typically staying 3–6 weeks in the hospital.

All patients at the department have a clinical case manager who coordinates their care (primary care, community services, and other secondary care) according to the Resource-group Assertive Community Treatment (R-ACT) model [23, 24]. A resource group consists of all supportive individuals in the patient’s network. Clinical as well as family/friends and others (e.g., representatives from community services or specialized care). Regular meetings in the patient’s resource group are organized by the case manager, and the patient chooses who to invite, typically the case manager, a psychiatrist, a representative from community services, and family/friend. These meetings are used to evaluate care and make plans that use the resources in the patient’s network. Several additional evidence-based interventions and evaluations are performed regularly (Table 1), complementing the resource group meetings. The case manager regularly conducts a health interview to assess the impact of life-style factors (e.g., diet, tobacco, alcohol, and exercise), to discuss health-related behaviors, and to jointly plan positive changes. Patients are offered annual follow-up visits with their clinical case managers to update patient background information and to assess eight diagnostic-specific core symptoms related to psychosis using the 8-item Positive and Negative Syndrome Scale (PANSS) [27] and to assess the patient’s reported level of functioning using the 12-item World Health Organization Disability Schedule (WHODAS 2.0). Patients should have regular visits to a psychiatrist to review medication as well as an annual somatic check-up.

**Table 1:** Schizophrenia care at the department. Seven activities, evidence-based interventions, and routine evaluations intended for all patients

1. Resource group meeting to assess and plan in the patient’s microsystem, including representatives (of the patient’s choice) from health and community care, friends, and family as well as others who have a supportive role in the patient’s life [25]. 2. Health interview to assess the impact of life-style factors, e.g., diet, tobacco, alcohol, and exercise and plan for interventions. 3. Patient background information to keep information up-to-date such as housing, children, and education. 4. Patient-reported 12-item WHODAS questionnaire to assess level of functioning [26]. 5. 8-item PANSS remission scale to assess eight diagnostic-specific core symptoms [27]. 6. Routine medication review. 7. Annual (somatic) check-up (e.g., weight, blood pressure, and blood samples).	

It is challenging for healthcare professionals to obtain an overview of what has been done related to interventions and routine evaluations for each patient and even more so when assessing whether a patient’s health has improved or declined. What is challenging for healthcare professionals is more or less impossible for patients as they have no way of accessing information on their health status in a user-adapted format. To address these challenges, the dashboard initiative was developed to provide information useful for patients and healthcare professionals at PoC that could be used to help assess, plan, and evaluate treatment and progress.

### Dashboard design and timeline

The dashboard was developed locally between 2015 and 2017, influenced by design thinking [28], loosely following a process to empathize, define, ideate, prototype, and test, moving back and forth between these stages. These design efforts fed into the process of creating a pilot platform that connected several tools, datasets, and visualization displays considered useful in psychosis care. The dashboard, designed to be used at the PoC, was central in the idea of supporting patients and professionals to jointly assess patient’s health, planning, and evaluation of care [21]. In parallel with designing the dashboard, joint work at the department and other psychiatric departments at the hospital resulted in developing a method to extract and structure relevant data from several information systems (e.g., patient records) into a useful data warehouse/model. Work processes related to data entry were redesigned to support more accurate and efficient documentation. A useful core dataset was defined in a process led by the management at the department, containing both process and outcome measures to be fed back regularly to users at different levels [29]. The dashboard was specifically designed to support collaborative evaluation of care at annual follow-up visits or more often if necessary.

### Content and functionality

Development of the dashboard aimed at supporting patients’ and clinicians’ co-production at the PoC by providing a joint interface to help

(i) assess progress by enabling the use of questionnaires, PROMs, a clinician-reported symptom/remission scale, and reporting results over time visualized in a graph,
(ii) improve outcomes by refining the content of treatment and care to adhere to recommended care by visualizing an automated checklist of evidence-based interventions and activities (e.g., resource group meeting, medication review, and annual check-up) (Box 1), and
(iii) minimize the administrative burden of having to document similar information in several different systems and registries.

**Fig. 1:**
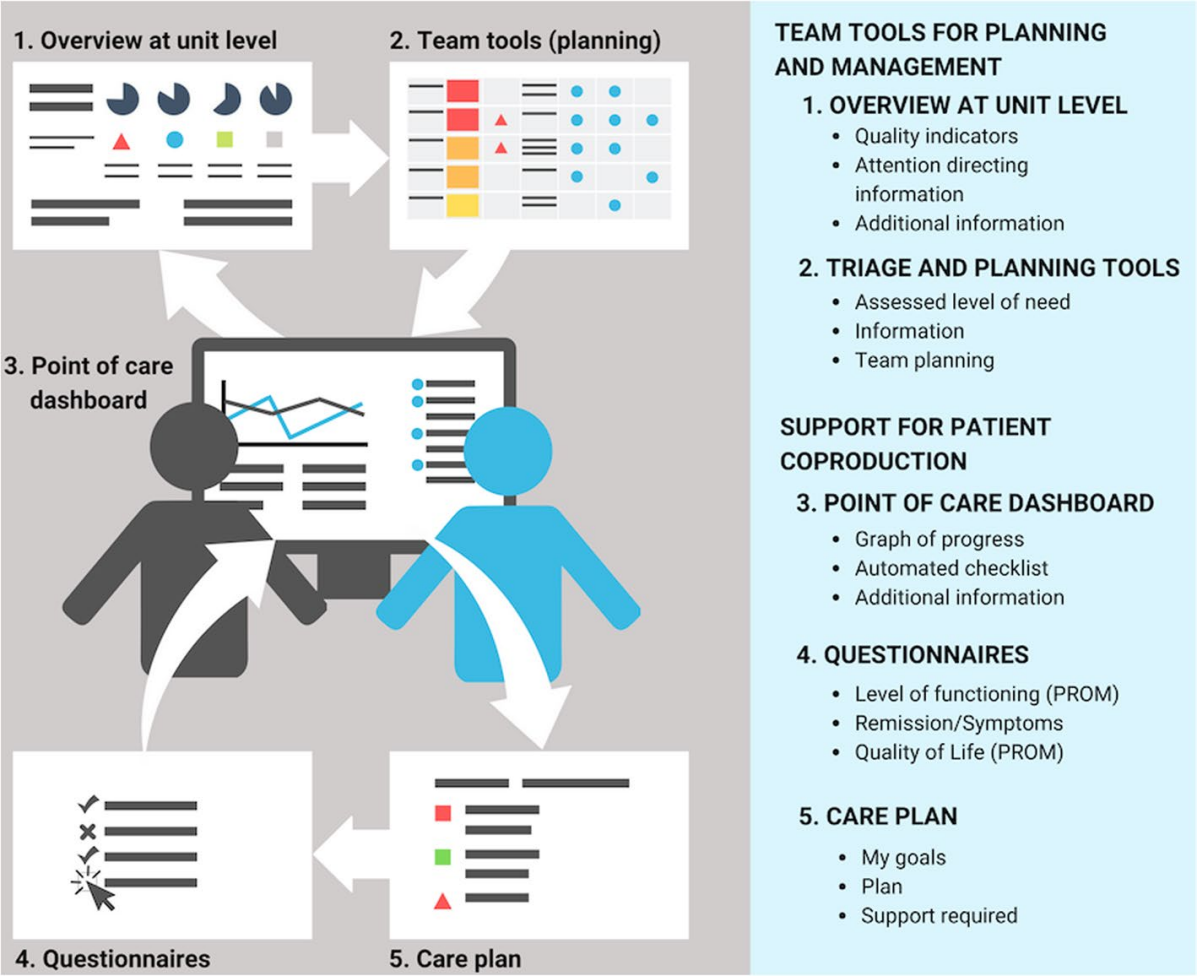
The Point-of-Care dashboard and related technologies described by Gremyr et al. [21]

The dashboard was one of several applications and displays developed to visualize data connected and fed by several information systems (Fig. 1) (see Gremyr et al. for details [21]). These applications include team tools for care planning and management such as a unit-level overview of quality indicators identifying patients at risk, triage and planning tools, the dashboard with its visualizations to support patient co-production, and applications supporting use of questionnaires (e.g., PROM) and treatment and care planning.

**Fig. 2:**
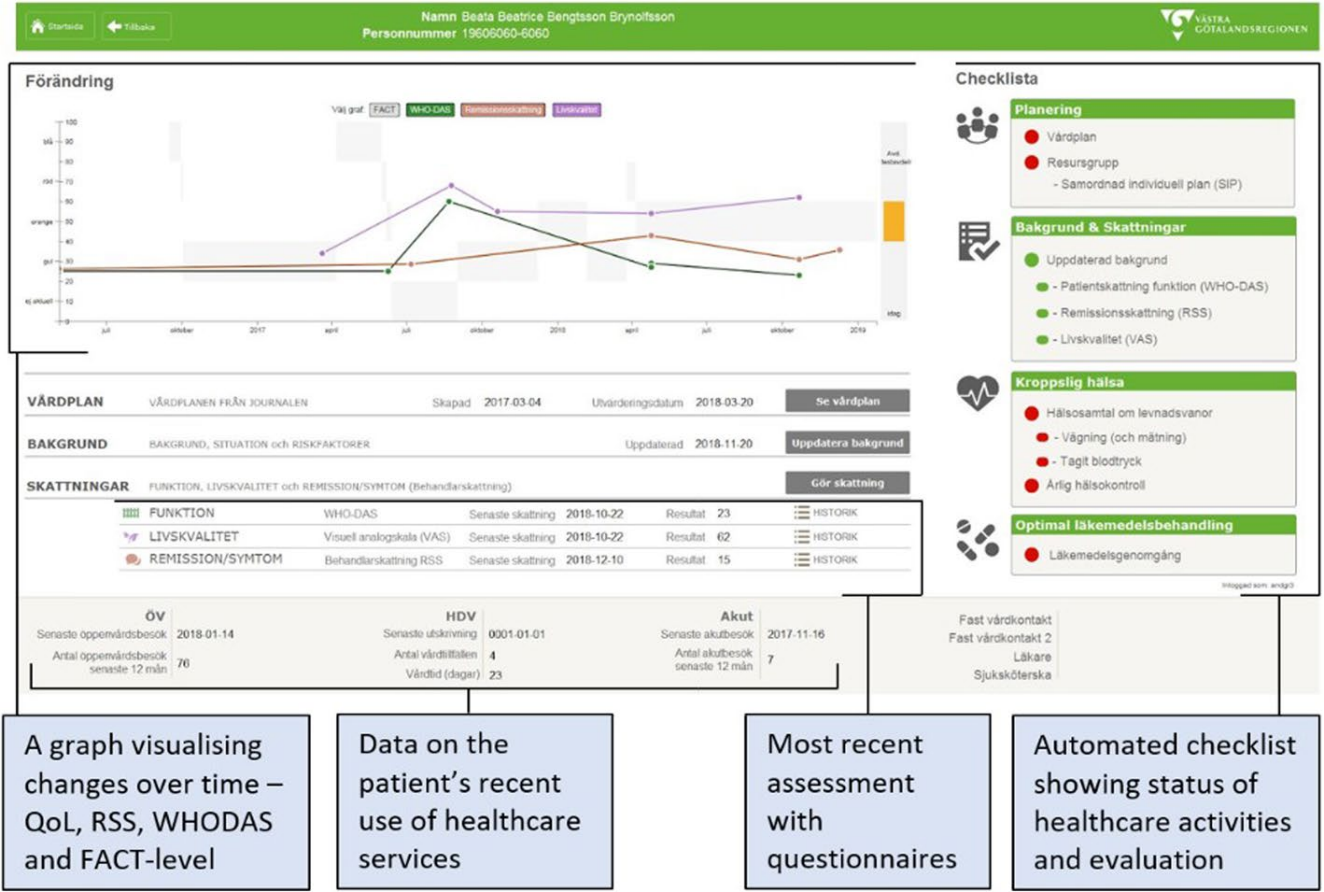
The dashboard showing progress in graphs as well as an automated checklist for a fictitious patient

Figure 2 illustrates the display, which visualizes changes over time and provides an automated checklist presenting the status of the patient’s healthcare interventions and activities. The display also presents evaluation data from the most recent assessments using questionnaires and data on the patient’s use of healthcare services (e.g., ER visits and admissions) (Fig. 2).

### The pilot tests

Pilot testing of the dashboard was initiated in August 2017 in an outpatient clinic serving 187 patients. Another outpatient clinic, serving 230 patients, started to use the dashboard in April 2018. The two pilot tests proceeded for 18 months each.

### The clinical adoption Meta model (CAMM)

**Fig. 3:**
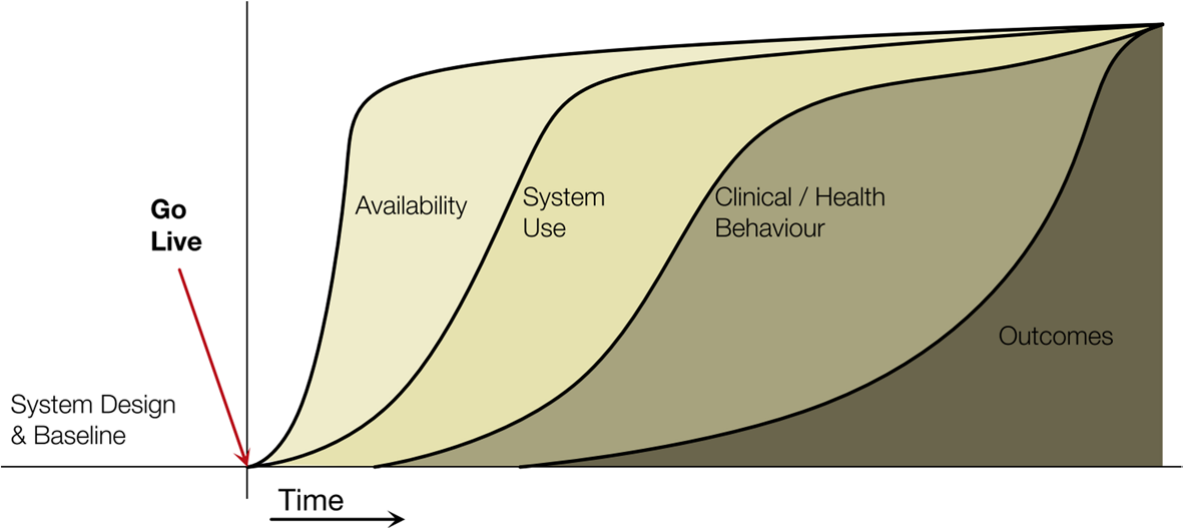
The four phases of the Clinical Adoption Meta Model [30]. Used with permission, CC BY 2.0

This evaluation of the dashboard pilot used the Clinical Adoption Meta Model (CAMM) to guide data collection and analysis. The CAMM was developed to support and evaluate the adoption and use of health information systems and apps, taking clinical benefit into account while addressing “the need to situate the evaluation throughout the adoption process, providing early and ongoing evaluation” [30 page 2]. The evaluation addresses four logically sequenced phases: accessibility, system use, clinical behavior, and clinical outcomes (Fig. 3).

*Accessibility or availability* refers to user access, system availability, and availability of content in the information system. These considerations address the extent users can access the system, content, and functionality. *System use* is defined as the interactions with the information system by intended end-users and refers to actual use (logins, time, and features used) and user experiences of the system. *Clinical (or health) behavior* is defined as meaningful adaptation of clinical processes or health behavior facilitated by the use of the information system. In other words, how and how much users change their behavior due to use of the information system. *Clinical outcomes* refers to the influence of the information system on behaviors and related outcomes.

### Data analysis

First, quantitative data from the health information system and data generated from the use of the dashboard by patients and clinicians as well as qualitative data from a focus group with clinicians using the dashboard were analyzed separately. Then, a convergent mixed-method design was applied [31] by merging analyses into one dataset to allow integrated reporting on the application and impact of the use of the dashboard, framed by the phases of CAMM.

### Quantitative data collection and analysis

Data on behavior were related to activities, evidence-based interventions, and regular evaluations intended for all patients as described in Box 1, with the ambition to regularly (i.e., at least annually) offer and deliver these to all patients.

The primary outcome measure was the patient-administered 12-item World Organization Disability Assessment Schedule 2.0 (WHODAS 2.0), which is used to assess functional impairment and disability [32]. The assessment was conducted during routine annual check-ups at the outpatient units. The Swedish version has been psychometrically validated at the department [26]. WHODAS captures an individual’s level of functioning with two items each representing six domains: Cognitive, Household, Society, Self-care, Mobility, and Social. Item scores range from 1 (no impairment) to 5 (extreme impairment). Therefore, the sum can range from 12 to 60 [26, 32]. Lower scores indicate less impairment and less disability.

The patients at the out-patient clinics using the dashboard were matched with patients at clinics not using the dashboard in terms of demographic characteristics: socioeconomic status of patients; proportion foreign born patients; and sex and age distributions. The non-dashboard clinics used paper forms for questionnaires and therefore did not have access to any visualizations of PROMs over time or other features of the dashboard (Fig. 2). Outliers were removed to create comparable groups in terms of the number of times the patients had conducted the WHODAS 2.0 (as a measure of exposure to intervention compared to treatment-as-usual). For all patients, we collected their first (t_1_) and most recent (t_2_) WHODAS 2.0 observation at the time of data collection (Fall, 2020) to compare the patient groups’ functioning (as an outcome indicator) and changes from t_1_ to t_2_.

Independent samples t-tests were used to compare the intervention and control groups’ mean age and mean number of days between WHODAS t_1_ and t_2_ since this data followed a normal distribution. Pearson chi-squared tests were used to compare the dashboard and non-dashboard groups’ gender proportion, to compare number of WHODAS observations per patient, and to compare the proportion of patients between the two patient groups that had lower (i.e., less function), the same, or higher (i.e., more function) impairment using WHODAS 2.0 scores between their first (t_1_) and most recent (t_2_) WHODAS observation. The statistical analyses were performed using SPSS (IBM corps., v.26), and *p* values of < 0.05 were considered statistically significant.

### Qualitative data and mixed-methods analysis

A convenience sample [33] was used when exploring case managers experiences of using the dashboard at point-of-care. The 25 case managers that had used the dashboard the most (out of 50 in total), were invited to participate in focus group interviews (FGI) planned at specific dates. Out of these, five case managers participated. The FGI was conducted and recorded digitally (Zoom Video Communications, Inc.), instead of on site, due to COVID-19 pandemic restrictions. The FGI was led by a moderator (co-author ACA) using a self-developed semi-structured interview guide with one assistant observer taking notes. The audio recording was transcribed verbatim and analyzed along with the observer’s notes using the CAMM model as a deductive frame [34, 35]. The transcripted data were matched by the emerging patterns relating to the CAMM model [35]. Pattern matching can be a good way to approach small qualitative material as pilot studies [35]. The analysis was first conducted independently by two authors; they discussed their differences until reaching an agreement. Finally, all the authors discussed the analysis until consensus was achieved. Thereafter, the descriptive quantitative data were sorted into the four phases, and similarities and differences were inferred [36]. The results are supported with quotations from the focus group interview (translated from Swedish to English by the authors).

## Results

The findings are presented in accordance with the CAMM framework. The four domains show similarities, data supporting each other, and differences. The different CAMM phases and the extent the dashboard was accessed, used, and associated with positive behavior changes and outcomes are presented.

### Access and availability

All 50 clinicians involved in the testing at the two pilot units accessed the system a total 4846 times between August 2017 and November 2019. No downtime was reported in the case management systems. Some participants, however, reported technical problems that interfered with accessing the system properly. When problems occurred, support was found to be helpful and swiftly available. However, this phase was of low concern to the focus group participants. Overall, the system availability, the content availability, and the user access were experienced to be high.

### System use

This domain refers both to use, as in number of logins and users per week, and to the participants’ experiences of use. Clinicians accessed the system on average 2.9 times per week. The analysis of dashboard use near the time of patient visits revealed that the dashboard was accessed for individual patients the same day as the patient visits to the clinic on about 300 occasions and about 200 times during the same hour patients visited. The dashboard was perceived to be of great support in keeping focus on process and progress both for individual patients and for the case managers in their work according to focus group participants. The use was primarily associated with positive experiences as reflected in the following quotations:

It is a fantastic tool, partly because of its utility in supporting value-creation for patients, and to audit our adherence to the treatment protocol. There’s such clarity.

Some patients said that there had not been any progress, but by showing the graphs, it made it possible to talk about the actual changes.

Participants reported both benefits and challenges using the dashboard. One identified benefit was how easy it was to access important information from several systems for planning and evaluation of treatments:

We could follow some metrics, e.g., BMI, blood pressure [.. .] days in hospital, weight, and remission. Instead of going into several systems, information is readily available in 5 seconds.

The use of the dashboard also entailed challenges. The level of cognitive functioning among patients was perceived to affect the extent patients were involved, which influenced their understanding of the usefulness of the dashboard:

It depends on the [patient’s] level of cognitive functioning. The higher the level, the easier it is to make use of it [the dashboard].

### Clinical or health behavior

Clinical or health behavior relates to both increase in productivity and to specific behavior changes. The department initiated large improvement initiatives related to evaluation efforts and increased use of data in reports. Yearly follow-ups and PROM measures were introduced and spread prior to and during the piloting of the dashboard. The dashboard was designed to support the tasks that every patient should be provided with yearly (see Box 1 for the interventions/activities). Data were reported to the outpatient units as a composite measure–i.e., the ratio of the unit’s patients who received the intervention/evaluation during the last 12 months–by showing a unit mean of the interventions (the evaluation index). This evaluation index could theoretically range from 0%, if no patients received any of the interventions/activities (Box 1) during the previous 12 months, to 100%, if all patients received all interventions/activities during the previous 12 months. All outpatient clinics increased the ratio of yearly follow-ups and use of PROMs during the time right before and during the pilots, with a greater increase at the beginning of the period. Clinics piloting the dashboard had higher evaluation indexes and increased more during the pilots than non-dashboard clinics. See Tables 2 a and b for evaluation indexes for the two clinics piloting the dashboard at start and end of pilot testing (after 18 months) compared to clinics not piloting the dashboard during the same period.

**Table 2:** a and b. The evaluation index at the clinics piloting the dashboard for 18 months compared to the mean evaluation index for clinics not using the dashboard

a		
Sept 2017–March 2019	at start	at end
Dashboard clinic 1, pilot	41.6%	58.5%
Non-dashboard clinics	27.3%	41.9%
b		
May 2018–November 2019	at start	at end
Dashboard clinic 2, pilot	54.4%	58.3%
Non-dashboard clinics	36.8%	39.8%

The participants in the focus group interview reported that the dashboard helped motivate both patients and case managers to continuously evaluate content and progress of care. It was found to be useful in supporting planning and following routines related to annual follow-ups. The dashboard increased the probability that patients received care according to the protocol and ensured that progress regarding patients’ health was identified:

[The dashboard] motivates patients to do a follow-up, they get back, [that’s] why we do it, and can see, just as we do, how things have progressed, and can plan ahead.

It could be challenging as clinicians to assess whether things had progressed or not, when in the middle of the conversation. But with this [the dashboard], it was possible to see that things actually had evolved and to address it differently.

The dashboard helped monitor and discover changes in outcomes, which prompted action that otherwise could have gone unnoticed. This helped the case managers be more attentive to patient’s needs and to address them more swiftly, sometimes by mobilizing the patient’s resource group:

Quality of Life has been like that [an indicator of how things change], and good to discuss in the resource groups. If it suddenly drops, most of the time something has happened.

When making use of the WHODAS results in the resource group, things can happen at once.

### Clinical outcomes

This domain is about patient, provider, organizational, and population outcomes and relates to clinical and health behavior changes as a result of using the dashboard. The analysis of the use of the dashboard cannot establish a clear cause-and-effect relationship. However, when comparing patient-reported function impairment between age- and sex-matched patients receiving care at the two comparable pilot units, a significantly higher proportion of patients at the units using the dashboard were improving (p = 0.045) (comparing first and most recent WHODAS sum score) (Table 3). For the sum score, 50% of patients at the units using the dashboard reported less function impairment at t_2_ than at t_1_ compared with 39% for the non-dashboard group.

**Table 3.**
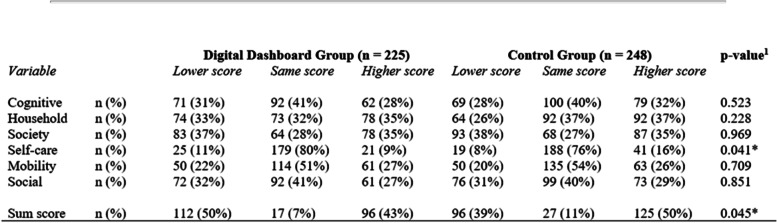
Differences in WHODAS 2.0 domain scores and sum scores between first (t_1_) and most recent (t_2_) observation between the intervention (dashboard users) and the control group (*N* = 473). Lower scores indicate improvement.

^1^Chi-squared test

*Significant at 0.05 level

Focus group participants mentioned that the dashboard supported their work and discussions in the resource group meetings, which was also confirmed by the differences in WHODAS 2.0 scores. In the domains (i.e., understanding and communicating, getting around, self-care, getting along, life activities, and participation in society), the dashboard group had a higher proportion of patients with improved functioning and a smaller proportion of patients with decreased functioning compared with the non-dashboard group in all domains, except participation in society. The differences were only statistically significant regarding self-care (*p* = 0.041), where only 9% of patients in the group using the dashboard reported more function impairment, compared with 16% in the control group (Table 3).

There were no significant differences in patients’ age (*p* = 0.445) or sex (*p* = 0.195) between the dashboard group (*n* = 225, x̄ age = 51.6, SD = 13.37, 44% females) and the non-dashboard group (*n* = 248, x̄ age = 52.6, SD = 13.98, 50% females).

Since the traditional mode of annual check-ups was established earlier than the testing of the dashboard, there was a slight difference in mean number of days (116) between the first (t_1_) and most recent (t_2_) WHODAS observations between the patient groups. However, there was no statistical difference in the number of WHODAS observations per patient between the group that had access to the dashboard (md = 2) and the group that did not have access to the dashboard (md = 2).

The dashboard supported data feedback-informed care aimed at improving patient health and social outcomes and using data to create understanding related to progress. Focus group participants reported that it was motivating for both patients and case managers to follow progress and to jointly celebrate when things were improving:

I recall, when showing improvements in outcomes–to share the joy, and for patients to see that we’re happy for them. To work in this way builds relationships.

The focus on improving outcomes for patients increased patient participation since the dashboard supports assessment of progress during visits. The use of PROM in the dashboard is considered a good tool for supporting goal setting and steering towards better outcomes by participants in the focus group:

It’s a good tool for clarifying a patient’s goals. Not the least to show what works.

Focus group participants reported mainly positive experiences using the dashboard as well as swifter responses to patient-reported needs and more adaptive responses to changes in a patient’s needs. However, they could not determine whether the use of the dashboard had helped improve patient-reported outcomes more than treatment-as-usual–i.e., before the introduction of the dashboard. This strengthens the notion that it is hard to assess progress over time when outcome measures are not used.

## Discussion

This study evaluates the use of the dashboard, particularly its utility and contribution to improved health for people with psychotic disorders such as schizophrenia. Several interesting findings were identified in this regard. First, the results related to access, use, behavior, and outcome showed that the pilot testing of the dashboard at the department appears to have an adoption archetype in line with “adoption with outcome benefits” [30]; that is, the system is accessed, used, and supports positive behavior changes that are related to improved outcomes over time.

The quantitative data showed that the use of the dashboard at PoC was positively associated with better patient-reported outcomes regarding overall level of functioning as well as self-care. The focus group interviews with case managers suggest that the dashboard helped include patients in assessments and evaluations as it allowed them to trace improvements (“a reason to celebrate”) and track sudden negative changes (“a reason to react”). This proactive stance is not possible to achieve if outcome measures cannot easily be monitored and understood jointly. This could possibly explain some of the relative increases in functioning observed in patients enrolled at the clinics that implemented the dashboard. Indeed, research suggests that under a recovery-oriented paradigm joint working between service users, clinicians, and family members in mental health is key to understanding and achieving patient empowerment, inclusion, and recovery [37].

The result supports the idea that the availability and use of the dashboard at PoC contributed to better health. The dashboard was mostly experienced as user friendly; if there were problems, support functions were in place. New technology needs to be easy to use and support co-production of care between care providers and patients [27].

Research has shown that patient participation can help improve the health of patients [13,14]. Easily available data used together with patients can improve co-production of care for persons suffering from severe mental illness (SMI) [38]. The dashboard’s ease of use conforms with the Swedish Patient Act, which requires that patients be given the opportunity to participate in their care [8]. The case managers found that some patients became more involved in their own care as the result of using the dashboard. The case managers also stressed that this depended on the patient’s cognitive status. Although some patient populations might have difficulties due to psychopathology and cognitive factors, this study found that the dashboard made it possible to co-produce care and even increase a sense of better self-care for individuals with psychotic disorders, which was the only WHODAS 2.0 function dimension that was statistically significant.

This study was conducted in close collaboration between the authors and the local hospital, which includes patients, professionals, managers, developers, and researchers. The interactive development can support easier use by healthcare professionals, since they feel more included in the work, which is important when dealing with complexity [21]. During this project, a major update of all computers within the healthcare organization made some central functionality (e.g., visualization of progress over time in a graph) not functional. Therefore, the patients were only included through the use of the WHODAS 2.0 PROM [22] and indirectly through reflections of their case managers in the focus group interview.

### Strengths and limitations

A strength of this study was the use of a mixed-method design for evaluation. Choosing a pragmatic approach, by mixing methods, offered us the potential for understanding the complexities and contexts of the dashboard in clinical practice. It also enhanced our capacity for social explanation and interpretation as we were informed by objective technical data (e.g., frequency of logins), PROM (WHODAS-2.0), and subjective experiences of the case managers (i.e., the focus group). Only a few case managers participated in the focus group interview, while being some of the most experienced regarding use of the dashboard, they cannot be assumed to be representative of all 50 users, but possibly most well prepared to give nuanced accounts of experience of the dashboards possibilities and challenges when used along with patients at point-of-care. The greatest limitation of this study is the lack of direct input from patients regarding the use and perceived usefulness of the dashboard at point of care in supporting coproduction and better health. Patients are represented through their PROMs and indirectly through the case managers’ experiences of interaction at point of care. Therefore, it makes this largely a study of the impact of clinicians using the PoC dashboard and its metrics, not necessarily only during patient encounters. This pragmatic approach, using mixed methods, helps us to more fully understand the clinical challenges and outcomes associated with the implementation of a new intervention. However, as mentioned by the originators of CAMM, Price and Lau [30], it does not allow us to establish a cause and effect relationship. Also, since it is set in a complex clinical setting including many factors, it is difficult to isolate if improvements are related to the active use of PROMs, use of the dashboard at point-of-care with patients, or other conditions. Thus, further investigations are needed to be able to establish causes and effects.

### Future research

Research suggests that clinician-patient relationships have a particular importance in psychiatry and that digital technologies might improve or challenge these relationships in different ways [39]. Therefore, patient perspectives are vital for understanding how we can enhance the clinician-patient encounter through a triangulated clinician-patient-technology collaboration. Future studies should interview patients to gain a deeper understanding of how they experience using visualization tools at PoC. This might increase the ability to assess and treat patients according to their current needs and to make early interventions if they are at risk of crisis.

## Conclusions

The case managers experienced the dashboard to be well functioning, user friendly, and impactful on their work as well as helpful for supporting patients’ health. This was also supported by PROM, which showed that patients at units using the dashboard experienced less function impairment over time compared with patients at units not using the dashboard. The dashboard collected and presented data in one interface, information that the health providers and patients could use together. This effect might have lowered the risk for missing changes in the patent health status and increased the ability to evaluate and treat patients according to their current needs.

## Supplementary Information

**Additional file 1.**

**Additional file 2.**

## Data Availability

The data used in the study were provided by the Sahlgrenska University Hospital. Authors of this study were granted rights to use the data, but not the right to share and distribute. Any reasonable request of data supporting this study in a disidentified and anonymous format, will be processed by the authors in collaboration with the Sahlgrenska University Hospital to ensure compliance with relevant laws, i.e. the Swedish data protection law and GDPR.

## Abbreviations

DALYs: Disability Adjusted Life Years
HER: Electronic Health Record
PoC: Point of Care
PROM: Patient-Reported Outcome Measures
SMI: Severe Mental Illness
GRAMMS: the Good Reporting of a Mixed Methods Study
WHODAS-2.0: World Organization Disability Assessment Schedule 2.0
PANSS: The Positive and Negative Syndrome Scale.
