# Supplementary material for: How a point-of-care dashboard facilitates co-production of health care and health for and with individuals with psychotic disorders: a mixed-methods case study
Source: BMC Health Serv Res. 2022 Dec 30;22:1599. doi: 10.1186/s12913-022-08992-2 (PMC9803257; doi:10.1186/s12913-022-08992-2)
Supplement: Supplementary file 1 — Additional file 1. [file 12913_2022_8992_MOESM1_ESM.docx]

# Appendix 1.

The prototypical adoption archetypes of the Clinical Adoption Meta Model by Price and Lau [[32]](https://paperpile.com/c/j8cFys/ICVro)

| The prototypical adoption archetypes | Description |
| --- | --- |
| 1. No Deployment. | Archetype describing a system that was not deployed. No access, use, behavior changes or outcomes traced. |
| 2. Low Adoption. | Availability of the system increases initially but does not lead to sustained use. No effects on behavior or outcomes. |
| 3. Adoption without Benefit (behavior and outcome). | A system that is accessed and used but the use does not lead to changes in behavior and outcomes. |
| 4. Behavior Change without Outcome Benefit. | A deployed system, accessed and used that supports changes in behavior but not resulting in changes in outcomes. |
| 5. Adoption with Outcome Benefits. | A system that is accessed, used, supporting sought for behavior changes leading to improved outcomes. |
| 6. Adoption with Harm. | A system, leading to negative behavior changes, and a negative effect on outcomes (harm). |
| 7. Benefit without Use. | A system that is accessed, but system use is low, and therefore cannot explain changes in behavior and outcomes. |
