# Supplementary material for: How a point-of-care dashboard facilitates co-production of health care and health for and with individuals with psychotic disorders: a mixed-methods case study
Source: BMC Health Serv Res. 2022 Dec 30;22:1599. doi: 10.1186/s12913-022-08992-2 (PMC9803257; doi:10.1186/s12913-022-08992-2)
Supplement: Supplementary file 2 — Additional file 2. [file 12913_2022_8992_MOESM2_ESM.docx]

# Appendix 2.

The GRAMMS Checklist from O´Cathain et al. [22]

| **Guideline** | **Section/Page** |
| --- | --- |
| Describe the justification for using a mixed method approach to the research question | Methods. Pg. 4 |
| Describe the design in terms of the purpose, priority and sequence of methods | Methods. Pg. 4, 10–11 |
| Describe each method in terms of sampling, data collection and analysis | Methods. Pg. 4, 8–11 |
| Describe where integration has occurred, how it has occurred and who has participated in it | Methods. Pg. 8–9 |
| Describe any limitation of one method associated with the present of the other method | Methods & Discussion. Pg. 9 & 19 |
| Describe any insights gained from mixing or integrating methods | Discussion. Pg. 19 |
